# Helicopter research: A persistent drawback to equitable collaborative research

**DOI:** 10.4314/gmj.v58i2.1

**Published:** 2024-06

**Authors:** Samuel Blay Nguah, Margaret Lartey

**Affiliations:** Department of Child Health, School of Medical Sciences, Kwame Nkrumah University of Science and Technology, Kumasi, Ghana; Department of Medicine & Therapeutics, University of Ghana Medical School, Accra, Ghana

## Introduction

Collaboration in research has increased over the years with many advances in medical knowledge. With understanding and good faith, many of these partnerships have yielded remarkable results and significantly improved our world. The inequity has resulted in many untoward effects since much of this collaborative research is between persons from richer or high-income countries and poorer or lower-income ones. Helicopter Research or “parachute research” refers to the situation where a more resourced partner in a research collaboration goes into the less resourced partner's location, conducts research, exports the data, and publishes the findings, often with no or very little input from or acknowledgement of the less resourced counterpart. This does not only occur between richer and poorer nations but also even within the same country where the relationship is asymmetrical.^1^

### Challenges with helicopter research

The effects of helicopter research are varied. First, local collaborators and communities are not or are minimally involved in the research planning, which is also of limited importance to them. The majority of such research does little to develop the local collaborators regarding individual skills acquisition, research resources, education, training and community development. Financial benefits are absent or very limited. The next significant effect of helicopter research is access to the research output. This includes access to human subject samples, data and research dissemination. Thus, the dominant partner decides which data is accessible and published. There may be no consultation, and the non-inclusion of locals as authors is common. It is well-established that publications from developed nations may have as low as 3% of their authors being Africans. In comparison, as much as 50% of African publications have co-authors who are not indigenes. In a review of articles published from Africa during the COVID-19 pandemic, one in five did not have an African author with 66% of all the authors not from the African continent.^2^

### Current solutions and shortcomings

Numerous attempts have been made over the past years to address the ills of helicopter research. In 2018, African scientists outlined how data and samples can be protected from exploitation.^3^ Also, the TRUST Code,^4^ authored by a thirteen-member research consortium, outlines twenty-three ways to reduce helicopter research.

These articles are extensive and deal with issues ranging from fairness, respect, care, and honesty in collaborative research. Unfortunately, the extent to which Institutional Review Boards (IRBs) implement these suggested remedies in these articles varies, limiting their effectiveness.

Some research sponsors require measures to enhance the benefits to the local communities as part of their terms of sponsorship; however, many sponsors do not consider these, making their sponsored research prone to exploitation. Furthermore, some IRBs now routinely consider issues aimed at limiting helicopter research. These include the insistence that the research proposal is considered only when there is a local collaborator or after local regulators have granted permission. In addition, most study processes should be conducted locally, or capacity should be built to conduct them locally where possible. Enforcement is yet to become worldwide.

At publication, the insistence of some journals on appropriate and adequate participation by local collaborators in any publication coming out of such collaborative research is now key.^5^ The Council for Science Editors (CSE) attempted to address this issue at its 2023 annual meeting through a symposium on journal policies for equitable research. The summary is published in the Science Editor.^6^ It addresses the role scientific journals could play in overcoming this challenge.

Some suggestions include having a well-written Memorandum of Understanding (MOU) between institutions, strengthening ethics committees to identify and reject helicopter research, and developing policies against the practice and enforcing them. Plos has developed a policy that it has started implementing, and Nature has a framework and code that it is also rolling out. Authors, publishers, funders, and institutes must work together to reduce this practice.

In summary, adapting and integrating the rather extensive TRUST Code into the functions of all IRBs will help check this effect. IRBs in high-income countries should collaborate and insist that local IRB directives be followed. Also, the role of permission from local authorities before the commencement of any research should be enforced. Community-based participatory research, a methodology whereby the community is treated not just as a subject but as an active participant and co-researcher, should be enhanced.^7^

Though this comes with difficulties, especially when the roles of the communities are not well defined, it will go a long way to counteract the ills of helicopter research.^8^ The routine addition of the caveat by funders that sponsored research should have as deliverables for local development, including funding for education, skills, and community development, should be the norm. More journals could emulate those that currently insist on including local collaborators as authors before publication to counteract the lopsided authorship that often characterises helicopter research effectively.

To conclude, helicopter research has long been within the research community. As awareness deepens and more organisations come on board, the stage is set for a paradigm change, and this must be whole and complete, integrating all solutions identified.
